# Warfarin maintenance dose prediction for Chinese after heart valve replacement by a feedforward neural network with equal stratified sampling

**DOI:** 10.1038/s41598-021-93317-2

**Published:** 2021-07-02

**Authors:** Weijie Ma, Hongying Li, Li Dong, Qin Zhou, Bo Fu, Jiang-long Hou, Jing Wang, Wenzhe Qin, Jin Chen

**Affiliations:** 1 Department of Evidence-Based Medicine and Clinical Epidemiology, School of Medicine/West China Hospital, Sichuan University, No. 17, Section 3, Renmin South Road, Chengdu, 610041 Sichuan China; 2 College of Computer Science, Sichuan University, Chengdu, Sichuan China; 3 Department of Cardiovascular Surgery, West China Hospital, Sichuan University, Chengdu, Sichuan China; 4 Department of Nutrition, The Second Affiliated Hospital of Chongqing Medical University, Chongqing, China; 5 Department of Cardiovascular Surgery, Tianjin Central Hospital, Tianjin, China; 6 Department of Career Development Division, The Fourth Affiliated Hospital of Anhui Medical University, Hefei, Anhui China; 7 Department of Social Medicine and Health Management, Shandong University, Jinan, Shandong China

**Keywords:** Data processing, Machine learning, Valvular disease

## Abstract

Patients requiring low-dose warfarin are more likely to suffer bleeding due to overdose. The goal of this work is to improve the feedforward neural network model's precision in predicting the low maintenance dose for Chinese in the aspect of training data construction. We built the model from a resampled dataset created by equal stratified sampling (maintaining the same sample number in three dose-groups with a total of 3639) and performed internal and external validations. Comparing to the model trained from the raw dataset of 19,060 eligible cases, we improved the low-dose group's ideal prediction percentage from 0.7 to 9.6% and maintained the overall performance (76.4% vs. 75.6%) in external validation. We further built neural network models on single-dose subsets to invest whether the subsets samples were sufficient and whether the selected factors were appropriate. The training set sizes were 1340 and 1478 for the low and high dose subsets; the corresponding ideal prediction percentages were 70.2% and 75.1%. The training set size for the intermediate dose varied and was 1553, 6214, and 12,429; the corresponding ideal prediction percentages were 95.6, 95.1%, and 95.3%. Our conclusion is that equal stratified sampling can be a considerable alternative approach in training data construction to build drug dosing models in the clinic.

## Introduction

Anticoagulant therapy aims to decrease the risk of thromboembolic events. Warfarin is the most widely prescribed oral anticoagulant for patients with heart valve prosthesis for its effectiveness and price comparing to new oral anticoagulants (e.g., dabigatran, rivaroxaban, apixaban, and edoxaban)^1–3^. Warfarin dosing is a daunting task due to its narrow therapeutic window and the complexity of dose influential factors (e.g., age, race, height, weight, smoking, the combination of drugs, intake of vitamin K in diet, and the polymorphism of CYP2C9 and VKORC1)^4–6^. Narrow treatment window and significant inter-individual variability lead to a tremendous difference in patients' dose requirements. The daily warfarin dose can differ by 20-fold between individuals^7^. Insufficient anticoagulation of warfarin can lead to thromboembolism, while excessive anticoagulation will cause bleeding events or even death^8^. No single warfarin dosing rule can guarantee its efficacy and safety. Individualized accurate dosing is needed to achieve the best clinical practice result.

Warfarin dose individualization aims to estimate the maintenance dose such that a patient maintains the international normalized ratio (INR) within the therapeutic range, and is traditionally achieved by an empirical warfarin dosing strategy that follows a heuristic process. In this clinical setting, working knowledge of the nonlinear relationship between warfarin dosage and INR response is required, which could also be non-trivial for physicians but can be possibly formulated as computational models.

Machine learning and artificial intelligence methods, in particular, artificial neural networks, have been making a significant impact on cardiovascular medicine^9,10^. Artificial neural networks are data-driven, i.e., network parameters are "learned" by a "training" process from a dataset. When a neural network has several layers, it can produce nonlinear representations of the underlying data structure. If sufficient retrospective records of patients' warfarin therapy history are available, it is possible to set up a computational model of neural networks to represent the relationship between the dose requirement and patients' characteristics.

Attempts have been made in the community to develop computer-aided warfarin dosing strategies and have shown promising results^11–14^. Existing approaches based on neural networks demonstrate their reliability in predicting warfarin maintenance doses in patients with medium-dose requirements but performed poorly for patients requiring a high dose and even worse for patients requiring a low dose^15–18^.

Neural network parameters are learned from data, and therefore the performance can be affected by the training dataset. In a typical clinical setting, the population of patients that require medium warfarin dose is often the largest, and the population requires a low dose is the smallest. Such a real-world dataset, if used directly, would possibly lead to neural network models that perform more accurately in the medium-dose range and less accurately in the low-dose range and high-dose range. Chinese are more sensitive to warfarin^19,20^. The precision of warfarin maintenance dose prediction is of great clinical significance, especially for patients whose requirements potentially lie in the low-dose range.

In this work, we assess the possibility of improving the low-dose warfarin prediction accuracy of a feedforward neural network by "designing" its training dataset. We build the training set via stratified random sampling; that is, we alter the dosage distribution in the training dataset such that the low, intermediate, and high daily dose are in equal proportions. Then we train a feedforward neural network by gradient descent using this resampled dataset. We detail our approach in “Methods”. Section “Results” is devoted to experimental results on a large multicentre database. A brief discussion and concluding remarks are given in “Discussion”.

## Methods

This study's protocol was approved by the Ethics Committee on Biomedical Research of West China Hospital of Sichuan University with the number 2020 (556). The study protocol was performed in accordance with the relevant guidelines. The informed consent was waived by the Ethics Committee on Biomedical Research of West China Hospital of Sichuan University with the number 2020 (556), given that this was a retrospective study.

### Participants

The raw data was from a multicentre (45 hospitals) database, "Chinese Low-Intensity Anticoagulant Therapy after Heart Value Replacement" (CLIATHVR), which consists of demographic information and regular anticoagulation monitoring records of 28,239 patients that underwent heart valve replacement and received warfarin anticoagulation therapy. The database was constructed and continuously updated from January 1st, 2011 to June 24th, 2016 (approved by the Ethics Committee of West China Hospital of Sichuan University with the number ChiECRCT-2011006).

We applied the following inclusion and exclusion criteria and used the resulting 19,060 patients' records.

Inclusion criteria: (1) age over 18 years; (2) after heart valve replacement (including bioprosthetic valve and mechanical valve); (3) receiving oral warfarin for anticoagulant treatment only and daily INR monitoring (measuring INR once a day); (4) obtaining maintenance dose (fluctuation range of INR value was less than 0.2 units for three times in succession and the therapeutic range was 1.5–2.5)^21^.

Exclusion criteria: (1) severe liver (the value of ALT or AST > 320 IU/L for male; ALT or AST > 224 IU/L for female) and kidney (the value of creatinine > 442 μmol/L or the value of urea nitrogen > 17.9 mmol/L) dysfunction before or after operation; (2) receiving aspirin, non-steroidal anti-inflammatory drugs, or other drugs affecting coagulation function; (3) suffering anticoagulation complications (e.g., thrombus, embolism, hemorrhage, and death) during anticoagulation therapy.

### Variable selection

We want to model the nonlinear relationship between warfarin maintenance dose and its influential factors. We applied the general linear model (GLM) Univariate procedure and used *P*-values and ${\eta }^{2}$ effect values as indicators to select potential and influential factors as input variables of the neural network model. The output variable was the warfarin maintenance dose, and its concrete values were identified when the fluctuation range of INR value is less than 0.2 units for three times in succession.

### Training datasets and validation dataset

In this study, we prepared four subsets, Set A, Set B, Set C and Set D. Set A and Set D were for neural networks training. Set B and Set C were for internal and external validation. We first divided the eligible 19,060 cases into three datasets: Set A, Set B, and Set C. Set C was a holdout dataset for validation and consists of the latest 1906 eligible cases recorded^22^. The remaining 17,154 eligible cases were randomly divided into Set A and Set B at ratio 8:1, i.e., Set A of 15,428 cases and Set B of 1906 cases, achieved in R studio (R Pack Version 3.6.3, R Studio, R Core Team, 2014, Boston, MA, USA). Set C was selected directly according to the latest enrolled time without randomizing to create a set containing patients with different features with Set A and B.

And then, we resampled the Set A to construct a training Set D. In China, the general dose of warfarin is 2.5 mg/day. According to the suggestions of clinicians and published researches, the cut-off values of dose were defined as 2.5 mg/day ± 0.25 × 2.5 mg/day (1.875 mg/day and 3.125 mg/day)^15,18^. We divided warfarin maintenance dose into three ranges, i.e., high-dose ≥ 3.125 mg/day, intermediate-dose 1.875–3.125 mg/day, and low-dose ≤ 1.875 mg/day. By following equal random stratified sampling, we kept all 1213 low-dose cases in Set A, and randomly resampled the intermediate-dose and the high-dose cases such that their numbers were reduced to be 1213. The resulting Set D consisted of 3639 cases.

### Model construction

In this study, neural networks were built by the backpropagation algorithm, and all have three layers, i.e., one input layer of n nodes, one hidden layer of m nodes, and one output layer of $l=1$ node. We denoted the neural networks trained from Set A as plain neural networks (PNNs) and those from Set D as stratified sampling trained neural networks (SSNNs).

The number $n$ is equal to the number of input variables. The number $m$ is set empirically

$$m=\surd nl+\alpha ,$$

where $\alpha \in ({0,10})$, a natural number, is a turning parameter.

The optimal $m$ and $\alpha$ are determined when we obtain the best prediction accuracy.

### Model validation

We compared the predicted warfarin maintenance dose with clinical data to demonstrate model performance. The comparing metrics were the mean absolute error (MAE), the mean square error (MSE), and the ideal prediction percentage (i.e., the absolute prediction error between predicted dose and the actual dose was within 20% of the actual dose).

### Statistical analysis

The *t* test was used to compare continuous variables in patient characteristics among different datasets, and the Wilcoxon rank-sum test was used when the variable did not meet the conditions of *t* test use. The χ^2^ test was used to compare categorical variables. The Monte Carlo method was adopted if the χ^2^ test criterion failed to meet (i.e., when more than 20% of the expected frequencies have a value of less than five, or the expected frequency was less than one). All statistical tests were two-sided, and *P* values less than 0.05 were considered statistically significant.

## Results

### Participant characteristics

There were 28,239 cases in the database, of which 19,060 were eligible. The participants were about 50 (50.65 ± 11.18) years old on average. Females account for 54.73%. Han Chinese, the dominant ethnic group in China, accounts for 96.07%. There were 15,336 patients taking an intermediate dose, which accounts for 80.5%. The low and high dose range covered 8.8% (1676 cases) and 9.7% (1848 cases), respectively (see Table S1 for details).

### Variable selection

We selected eight factors as input variables ($n=8$) from 51 potential independent factors (listed in Table S1) to build the model after applying the GLM Univariate procedure ($P<0.05,{\eta }^{2}\ge 0.002$). The eight factors were initial dose (${\eta }^{2}=0.027$), albumin (${\eta }^{2}=0.002$), creatinine (${\eta }^{2}=0.003$), activated partial thromboplastin (APPT, ${\eta }^{2}=0.002$), starting time of anticoagulation (${\eta }^{2}=0.002$), type of disease (${\eta }^{2}=0.002)$, tricuspid valve disease (${\eta }^{2}=0.002)$, and method of initial dosing (${\eta }^{2}=0.004$).

The output variable was warfarin maintenance dosage.

There were no statistical differences among the input variables in Set A and B ($P>0.05$), which is expected as cases in these two sets are all sampled randomly.

The differences in all the selected input variables, except APPT, were statistically significant in comparing Set C with Set A ($P\le 0.005$). This is expected as all 1906 cases in Set C are in chronological order and were set aside from the eligible cases. And thus, we assume that Set C is representative and would sufficient for model validation. (More statistical details on the data are listed in Table 1).

**Table 1:** Statistics on the factors in the training, internal validation, and external validation dataset (i.e., Set A, B, and C).

Characteristic (unit)	Training set (N = 15,248)	Internal validation set (N = 1906)		External validation set (N = 1906)	
	Mean ± SD/N (%)	Mean ± SD/N (%)	*P*-value	Mean ± SD/N (%)	*P*-value
Initial dose (mg/day)	2.93 ± 0.72	2.93 ± 0.70	1.00	2.66 ± 0.52	< 0.001
Albumin (g/L)	41.59 ± 4.42	41.45 ± 4.44	0.19	41.29 ± 4.24	0.005
Creatinine (μmol/L)	77.26 ± 19.15	76.84 ± 19.02	0.37	80.81 ± 20.29	< 0.001
APPT (s)	31.21 ± 7.65	31.55 ± 7.37	0.067	30.89 ± 5.71	0.077
Starting time of anticoagulation (X days after surgery) (days)^a^	2	2	0.26	2	< 0.001
**Type of disease**			0.24		< 0.001
Rheumatic heart disease	12,664 (83.05)	1573 (82.53)		1563 (82.00)	
Degenerative mitral valve disease	617 (4.05)	79 (4.14)		60 (3.15)	
Degenerative aortic valve disease	970 (6.36)	133 (6.98)		87 (4.56)	
Congenital heart disease	13 (0.09)	0		0	
Degenerative cardiac conduction system disease	564 (3.70)	72 (3.78)		87 (4.56)	
Ischemic heart disease	17 (0.11)	6 (0.31)		1 (0.05)	
Infective endocarditis	204 (1.34)	15 (0.79)		39 (2.05)	
Secondary valvular heart disease	89 (0.58)	12 (0.63)		36 (1.89)	
Traumatic valvular heart disease	8 (0.05)	0		0	
Dilated cardiomyopathy	3 (0.02)	0		0	
Hypertrophic cardiomyopathy	9 (0.06)	1 (0.05)		4 (0.21)	
Systemic autoimmune disease	90 (0.59)	15 (0.79)		29 (1.53)	
**Tricuspid valve disease**			0.34		< 0.001
Stenosis	165 (1.08)	21 (1.11)		20 (1.05)	
Insufficiency	7610 (49.91)	993 (52.10)		1087 (57.03)	
Stenosis and insufficiency	137 (0.90)	17 (0.89)		18 (0.94)	
**Method of initial dosing**			0.12		< 0.001
Saturated dose	2647 (17.36)	304 (15.95)		70 (3.67)	
General dose	12,601 (82.64)	1602 (84.05)		1836 (96.33)	

*APPT* activated partial thromboplastin time, *SD* standard deviation, *Saturated dose* dose ranging from 5 to 10 mg/day; General dose, dose ranging from 2.5 to 5 mg/day.

^a^Starting time of anticoagulation (X days after surgery) (days) were showed by median because of its right skewed distribution. Continuous variables materials were analyzed by using independent sample *t* test; Categorical data materials were analyzed by using chi-square analysis. The starting time of anticoagulation was analyzed by using Wilcoxon rank-sum test; The type of disease was analyzed by using Monte Carlo method.

### Model construction

**Figure 1:**
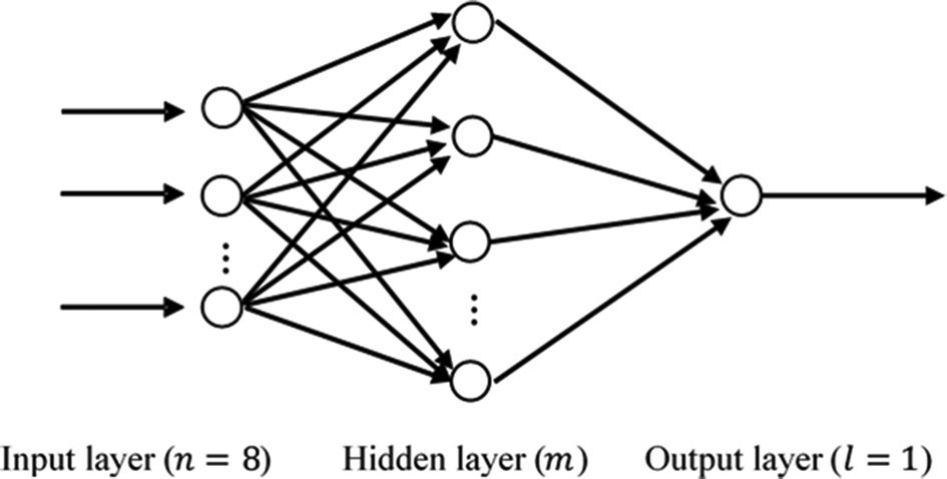
The structure of the feedforward models in this study. The single input layer has $n=8$ nodes. The output layer has $l=1$ node. The hidden layer has $m$ nodes; the concrete values of $m$ in the models—PNN, SSNN, PNNlow-dose, PNNhigh-dose, PNNmid-dose (N = 12,429), PNNmid-dose (N = 6214), PNNmid-dose (N = 1533)—were 5, 9, 11, 12, 7, 7, and 10.

The learning rate, expected error, and training times were tuned to be 0.1, 0.001, and 1000, respectively. The final model structure (both for PNNs and SSNNs) is shown in Fig. 1. All neural network models built in our study had eight inputs ($n=8$) and one output ($l=1$); the optimal values of $m$, the number of nodes of the hidden layer, varied across different models (see Fig. 1 for their concrete values).

The PNN model was built based on Set A (15,248 cases) and the SSNN model was built based on Set D (3639 cases) containing the 1213 cases in each dose group.

### Model validation

The difference between internal validation dataset B and the external validation dataset C were not significant in either MAE or MSE. On dataset B, the difference of ideal predicted percentage between PNN and SSNN was not significant, nor did the difference of MSE or MAE, and all of them were less than 0.5 mg/day. Similar results were also observed on dataset C. The resampling process that we performed reduced the training dataset's size, but did not greatly affect the overall predicting precision (see Table 2).

**Table 2:** Overall predictive accuracy comparison: PNN vs. SSNN.

Validation set	Model	MAE (mg/day)	Under-predicted percentage N (%)	Ideal-predicted percentage N (%)	Over-predicted percentage N (%)	MSE (mg/day)
Internal	PNN	0.3250	159 (8.3)	1507 (79.1)	240(12.6)	0.3475
	SSNN	0.4341	185 (9.7)	1438 (75.4)	292 (14.9)	0.4230
External	PNN	0.3452	99 (5.2)	1456 (76.4)	351 (18.4)	0.3933
	SSNN	0.4108	154 (8.1)	1441 (75.6)	311 (16.3)	0.4085

**Figure 2:**
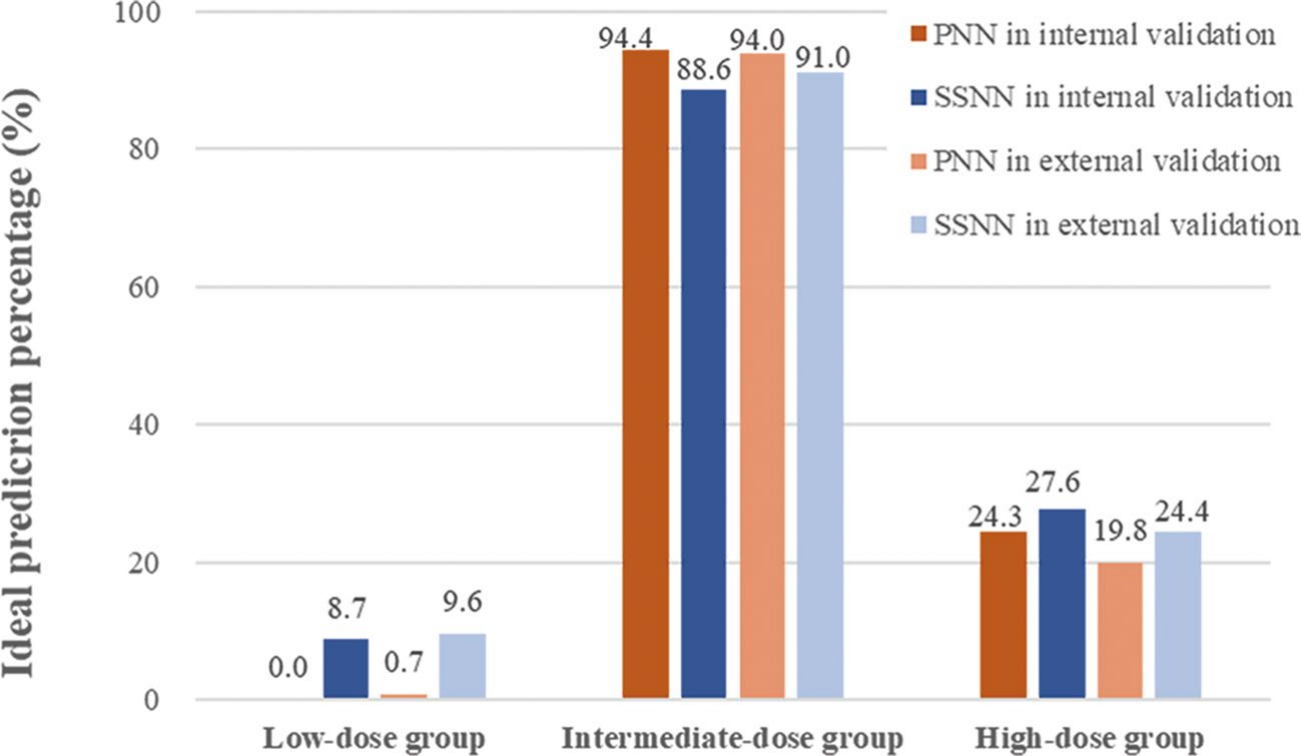
Predictive performance of PNN and SSNN in terms of ideal prediction percentage on internal and external dataset.

A closer look at these results on Set B and C revealed that: (1) when cases lay in the medium-dose range, both PNN and SSNN predicted the best (ideal predicted percentage were around 90%); (2) when cases lay in the low-dose range, the dose predicted by PNN was overestimated (the prediction accuracy was 0.0% on Set B and 0.7% on Set C), and SSNN performed better than PNN (the prediction accuracy was 8.7% on Set B and 9.6% on Set C); (3) when cases lay in the high-dose range, prediction by SSNN was also superior, and there was an improvement in prediction, e.g., from 24.3 to 27.6% on Set B and from 19.8 to 24.4%. See Table 3 and Fig. 2 for details.

**Table 3:** Predictive accuracy comparison: PNN vs. SSNN on sub-dose groups.

Validation set	Model	Low-dose group (≤ 1.875 mg/day)			Intermediate-dose group (1.875–3.125 mg/day)			High-dose group (≥ 3.125 mg/day)		
		Under (%)	Ideal (%)	Over (%)	Under (%)	Ideal (%)	Over (%)	Under (%)	Ideal (%)	Over (%)
Internal	PNN	0 (0.0)	0 (0.0)	172 (100.0)	19 (1.2)	1462 (94.4)	68 (4.4)	140 (75.7)	45 (24.3)	0 (0.0)
	SSNN	0 (0.0)	15 (8.7)	157 (91.3)	56 (3.6)	1372 (88.6)	121 (7.8)	129 (69.7)	51 (27.6)	5 (2.7)
External	PNN	0 (0.0)	2 (0.7)	289 (99.3)	30 (2.0)	1437 (94.0)	62 (4.0)	69 (80.2)	17 (19.8)	0(0.0)
	SSNN	1 (0.3)	28 (9.6)	262 (90.1)	88 (5.8)	1392 (91.0)	49 (3.2)	65 (75.6)	21 (24.4)	0(0.0)

We further trained more PNNs individually on single-dose subsets of the eligible cases. The training sets of these models were originated from single-dose subset and thus each training set containing patients from a single dose group only. Specifically, for the low-dose subset and the high-dose subset, the training sample numbers were 1340 and 1478, and the ideal prediction percentages were around 70% (66.7% and 73.5% in internal validation; 70.2% and 75.1% in external validation). For the intermediate-dose, we trained three PNN models separately from 1553, 6214, and 12,429 samples and found that the ideal prediction percentages were more than 95% in both internal and external validation (see Tables 4 and 5). These findings suggested that the training samples were adequate and the selected factors in building the models in this study were appropriate.

**Table 4:** Predictive accuracy of PNN models trained from samples in single dose range.

Validation set	Training set (dose range, sample number)	MAE (mg/day)	Under-predicted percentage N (%)	Ideal-predicted percentage N (%)	Over-predicted percentage N (%)	MSE (mg/day)
Internal	Low, N = 1340	0.2597	18 (10.7)	112 (66.7)	38 (22.6)	0.1051
	Intermediate, N = 1533	0.0813	12 (0.0)	6014 (96.5)	189 (3.5)	0.0282
	High, N = 1478	0.5467	22 (11.9)	136 (73.5)	27 (14.6)	0.5317
External	Low, N = 1340	0.2500	2 (1.2)	118 (70.2)	48 (28.6)	0.0988
	Intermediate, N = 1533	0.0844	15 (0.1)	7424 (95.1)	329 (4.8)	0.0327
	High, N = 1478	0.6038	18 (9.7)	139 (75.2)	28 (15.1)	0.5996

**Table 5:** Predictive accuracy of PNN models trained from different number of intermediate-dose samples.

Validation set	Training set size	MAE (mg/day)	Under-predicted percentage N (%)	Ideal-predicted percentage N (%)	Over-predicted percentage N (%)	MSE (mg/day)
Internal	12,429	0.0681	0 (0.0)	1488 (95.8)	66 (4.2)	0.0281
	6214	0.0665	0 (0.0)	4497 (96.5)	164 (3.5)	0.0256
	1533	0.0813	12 (0.2)	6014 (96.8)	189 (3.0)	0.0282
External	12,429	0.0830	0 (0.0)	1481 (95.3)	73 (4.7)	0.0310
	6214	0.0690	4 (0.1)	4433(95.1)	224 (4.8)	0.0305
	1533	0.0844	15 (0.2)	7424 (95.6)	329 (4.2)	0.0327

## Discussion

In this work, we have assessed the predictive performance of a neural network built from a stratified training dataset for low-dose warfarin therapy. We have shown that the dose distribution of the training cases can affect the low-dose predictive performance of a feedforward neural network. When the training dataset was randomly stratified into the three subgroups (i.e., the low-dose, intermediate-dose, and high-dose) and all contain the same number of cases, the low-dose predictive performance of the neural network increased from 0.0 to 8.7% on internal validation and from 0.7 to 9.6% on external validation. This suggests that the feedforward neural network can predict low-dose warfarin requirements more accurately by resampling its training dataset.

Several works have demonstrated the possibility that algorithms basing on machine learning can predict warfarin dose requirements more precisely^18,23–25^. However, given that their study numbers are relatively small, the patients might not represent all warfarin users^15,24,25^. These works also usually focus on the overall predicting performance, with limited further discussion on subgroups. Neither did they perform external validation^24,25^. The studies by Qian et al.^23^ and Tao et al.^18^ indicate that predictive accuracy can be different across subgroups, whereas no specific solution was mentioned.

We carried out equal random stratified sampling on the dataset to obtain the training data^26^. We observed the improvement in low-dose predictive performance (there was also an increase in the high-dose subgroup), while the alternation in the data distribution also affected the predictive precision of the intermediate-dose subgroup with a loss of 3.0%. This dropping was possibly caused by the reduction in the training set. However, additional experiments carried out separately on each subgroup demonstrated that a training set of over 1000 cases was sufficient to obtain a high predictive precision (e.g., 95.6% with 1553 cases. see Tables 4 and 5 for more details), which also meant that the eight factors characterize patients well for warfarin maintenance dose prediction, especially for cases in the intermediate-dose range.

We further compared the three models trained on the three subgroups with the same relatively small number of cases. We noticed that all three models predict more precisely, and the predictive performance of the model trained on the intermediate-dose cases was better than the other two. We expect that a model built via multi-task learning would perform better in terms of overall prediction accuracy and subgroups dosing precision.

It is reported that genes CYP2C9 and VKORC1 can account for about 40% of the variation in warfarin dose requirements^27^. The International Warfarin Pharmacogenetics Consortium (IPWC) dosing algorithm also indicated that genetic data could improve a model's predictive performance^28^. We assume that involving genotype factors may further improve our predicting precision for the low-dose and high-dose cases, since genotype difference could be a primary contributor to the nonlinearity of the INR dose–response curve. At the time of this study, we did not have sufficient cases with genotype information; further works are needed to investigate whether genotypes have an equal effect on these three dose ranges.

Besides genotypes, we did not consider dietary factors either, for the difficulty in data collection and the vast regional diet difference across the regions where the 45 hospitals locate. The database lacked these factors. However, this kind of data reflects the typical patients in China, where cost-effectiveness is also an important issue to consider^29,30^. Some factors such as age, gender, and weight are of importance in clinic medicine. We initially considered and added age, gender, height, weight, ALT, and AST to the model, but founded that it did not improve the predictive effects obviously; we provided more experimental details on this issue in Table S2. For the sake of model simplicity, we did not include these factors in the model. Our data-driven model could be a supplement to clinical experience for clinicians in medication decision making. For limitations in the modeling, we restricted the neural networks to be three-layered for simplicity; we have not fully explored multi-layer structure, multi-task learning^31^, nor other neural network models. Drug interaction is a critical factor to influence the INR change. To explore the warfarin anticoagulation therapy suitable for Chinese and eliminate the influence of other drugs affecting the efficacy judgement, the database excluded the patients who received the drugs affecting coagulation function and collected few information of drug interaction, so we did not include this factor in the model. We would collect the data of drug interaction in our future study.

Our study indicated that the warfarin dose distribution in the training data affected a neural network's predictive performance on the low-dose group. We expect that a genotype-guided version of our approach would predict more precisely. Further work is also needed to explore whether performance can be improved by an additional increase in the low-dose and high-dose population, e.g., through a repeated randomly stratified sampling process. Recently works on generative adversarial networks also indicate another possibility for data augmentation^32^.

## Supplementary Information

Supplementary Table S1.

Supplementary Table S2.
