# Supplementary material for: Warfarin maintenance dose prediction for Chinese after heart valve replacement by a feedforward neural network with equal stratified sampling
Source: Sci Rep. 2021 Jul 2;11:13778. doi: 10.1038/s41598-021-93317-2 (PMC8253817; doi:10.1038/s41598-021-93317-2)
Supplement: Supplementary file 1 — Supplementary Table S1. [file 41598_2021_93317_MOESM1_ESM.docx]

Warfarin maintenance dose prediction for Chinese after heart valve replacement by a feedforward neural network with equal stratified sampling

Weijie Ma^1,†^, Hongying Li^2,†^, Li Dong^3^, Qin Zhou^4^, Bo Fu^5^, Jiang-long Hou^3^, Jing Wang^6^, Wenzhe Qin^7^ and Jin Chen^1,^*

^1^ Department of Evidence-based Medicine and clinical epidemiology, School of Medicine/West China Hospital, Sichuan University, Chengdu, Sichuan, China

^2^ College of Computer Science, Sichuan University, Chengdu, Sichuan, China

^3^ Department of Cardiovascular Surgery, West China Hospital, Sichuan University, Chengdu, Sichuan, China

^4^ Department of Nutrition, the second affiliated hospital of Chongqing medical university, Chongqing, China

^5^ Department of Cardiovascular Surgery, Tianjin central hospital, Tianjin, China

^6^ Department of Career development division, the fourth affiliated hospital of Anhui Medical University, Hefei, Anhui, China

^7^ Department of Social Medicine and Health Management, Shandong University, Jinan, Shandong, China

Corresponding author: Jin Chen, ebm_chenjin@126.com

610041 No. 17, Section 3, Renmin South Road, Chengdu

Tel: (028) 8542-2082

† these authors contributed equally to this work

* Corresponding author

**Table S1** Patients’ basic clinical characteristics

| **Characteristic(unit)** | Total cases (N=19060)  N (%) / mean ± SD |
| --- | --- |
| **Gender (female)** | 10432 (53.73%) |
| **Age (year)** | 50.65 ± 11.18 |
| **Ethnic group (Han)** | 18310 (96.07%) |
| **Height (cm)** | 162.72 ± 8.19 |
| **Weight (kg)** | 60.66 ± 10.90 |
| **BSA (m^2^)** | 1.62 ± 0.17 |
| **BMI (kg/m^2^)** | 22.84 ± 3.27 |
| **Left ventricular diastolic diameter (mm)** | 57.09 ± 8.85 |
| **Inner diameter of left atrium (mm)** | 49.41 ± 12.75 |
| **Inner diameter of right atrium (mm)** | 38.54 ± 13.42 |
| **Inner diameter of right ventricular (mm)** | 26.04 ± 8.79 |
| **EF (%)** | 58.67 ± 8.86 |
| **Type of disease** |  |
| Rheumatic heart disease | 15800 (82.90%) |
| Degenerative mitral valve disease | 756 (3.97%) |
| Degenerative aortic valve disease | 1190 (6.24%) |
| Congenital heart disease | 13 (0.07%) |
| Degenerative cardiac conduction system disease | 723 (3.97%) |
| Ischemic heart disease | 24 (0.13%) |
| Infective endocarditis | 258 (1.35%) |
| Secondary valvular heart disease | 137 (0.72%) |
| Traumatic valvular heart disease | 8 (0.04%) |
| Dilated cardiomyopathy | 3 (0.02%) |
| Hypertrophic cardiomyopathy | 14 (0.07%) |
| Systemic autoimmune disease | 134 (0.70%) |
| **Atrial fibrillation history** | 5504 (28.88%) |
| **Cardioversion** | 93 (0.49%) |
| **Embolism history** | 205 (1.08%) |
| **Thrombus** | 738 (3.87%) |
| **Bleeding disease history** | 147 (0.77%) |
| **History of anticoagulant drugs** | 597 (3.13%) |
| **Hypertension history** | 2337 (12.26%) |
| **Diabetes history** | 611 (3.21%) |
| **Operation history** | 2126 (11.15%) |
| **ALT (IU/L)** | 25.02 ±17.89 |
| **AST (IU/L)** | 25.77 ± 13.58 |
| **Total albumen (g/L)** | 68.61 ± 6.75 |
| **Albumin (g/L)** | 41.55 ± 4.41 |
| **Albumin / Globulin** | 1.61 ± 0.74 |
| **Urea nitrogen (mmol/L)** | 6.14 ± 2.02 |
| **Creatinine (μmol/L)** | 77.57 ± 19.29 |
| **PT (s)** | 13.02 ± 2.90 |
| **APPT (s)** | 31.21 ± 7.45 |
| **Preoperative INR** | 1.10 ± 0.27 |
| **NYHA classification** |  |
| Ⅰ class | 229 (1.20%) |
| Ⅱ class | 5097 (26.74%) |
| Ⅲ class | 13126 (68.87%) |
| Ⅳ class | 608 (3.19%) |
| **ECG** |  |
| Sinus rhythm | 11521 (60.45%) |
| Atrial fibrillation | 7396 (38.80%) |
| Atrial flutter | 143 (0.75%) |
| **Mitral disease** |  |
| Stenosis | 3761 (19.73%) |
| Insufficiency | 4780 (25.08%) |
| Stenosis complicated with insufficiency | 6615 (34.71%) |
| **Tricuspid valve disease** |  |
| Stenosis | 206 (1.08%) |
| Insufficiency | 9690 (50.84%) |
| Stenosis complicated with insufficiency | 172 (0.90%) |
| **Aortic disease** |  |
| Stenosis | 1356 (7.12%) |
| Insufficiency | 7339 (38.50%) |
| Stenosis complicated with insufficiency | 4415 (23.16%) |
| **Mitral valve surgery** |  |
| Plastic | 524 (2.75%) |
| Replacement | 13175 (69.12%) |
| **Tricuspid valve surgery** |  |
| Plastic | 7091 (37.20%) |
| Replacement | 360 (1.89%) |
| **Aortic valve surgery** |  |
| Plastic | 79 (0.40%) |
| Replacement | 10122 (53.12%) |
| **Pulmonary valve surgery** |  |
| Plastic | 16 (0.08%) |
| Replacement | 24 (0.13%) |
| **Left atrial appendage treatment** |  |
| Non-treated | 17585 (92.26%) |
| Ligation | 859 (4.51%) |
| Excision | 198 (1.04%) |
| Internal suturing | 418 (2.19%) |
| **Thrombus removal** | 1386 (7.27%) |
| **Radiofrequency ablation** | 1604 (8.42%) |
| **Left atrium reduction** | 496 (2.60%) |
| **Origin of warfarin (China)** | 9890 (51.89%) |
| **Warfarin manufacturer** |  |
| Finland | 9898 (51.93%) |
| Shandong (China) | 4806 (25.22%) |
| Shanghai (China) | 4327 (22.70%) |
| Others | 29 (0.15%) |
| **Dosage form** |  |
| 2.5mg/tablet | 9237 (48.46%) |
| 3mg/tablet | 9823 (51.54%) |
| **Method of initial dosing** |  |
| Saturated dose | 3021 (15.85%) |
| General dose | 16039 (84.15%) |
| **Starting time of anticoagulation (X days after surgery) (d)*** | 2 |
| **Initial dose (mg/d)** | 2.91 ± 0.70 |
| **Warfarin maintenance dose (mg/d)** | 2.74 ± 0.69 |
| **Warfarin maintenance dose subgroups** |  |
| low-dose | 1676 (8.79%) |
| intermediate-dose | 15536 (81.51%) |
| high-dose | 1848 (9.70%) |

SD, standard deviation; BSA, body surface area=0.0061×height (cm)+0.0128×weight (kg)-0.1529; BMI, body mass index; EF, ejection fraction; ALT, alanine transaminase; AST, aspartate aminotransferase; APTT, activated partial thromboplastin time; INR, International normalized ratio; ECG, electrocardiograph; NYHA, New York Heart Association; Saturated dose, dose ranging from 5mg/d to 10mg/d; General dose, dose ranging from 2.5mg/d to 5mg/d.

*Starting time of anticoagulation (X days after surgery) (d), shown by median (because its distribution is right skewed).
