# Supplementary material for: Warfarin maintenance dose prediction for Chinese after heart valve replacement by a feedforward neural network with equal stratified sampling
Source: Sci Rep. 2021 Jul 2;11:13778. doi: 10.1038/s41598-021-93317-2 (PMC8253817; doi:10.1038/s41598-021-93317-2)
Supplement: Supplementary file 2 — Supplementary Table S2. [file 41598_2021_93317_MOESM2_ESM.docx]

Warfarin maintenance dose prediction for Chinese after heart valve replacement by a feedforward neural network with equal stratified sampling

Weijie Ma^1,†^, Hongying Li^2,†^, Li Dong^3^, Qin Zhou^4^, Bo Fu^5^, Jiang-long Hou^3^, Jing Wang^6^, Wenzhe Qin^7^ and Jin Chen^1,^*

^1^ Department of Evidence-based Medicine and clinical epidemiology, School of Medicine/West China Hospital, Sichuan University, Chengdu, Sichuan, China

^2^ College of Computer Science, Sichuan University, Chengdu, Sichuan, China

^3^ Department of Cardiovascular Surgery, West China Hospital, Sichuan University, Chengdu, Sichuan, China

^4^ Department of Nutrition, the second affiliated hospital of Chongqing medical university, Chongqing, China

^5^ Department of Cardiovascular Surgery, Tianjin central hospital, Tianjin, China

^6^ Department of Career development division, the fourth affiliated hospital of Anhui Medical University, Hefei, Anhui, China

^7^ Department of Social Medicine and Health Management, Shandong University, Jinan, Shandong, China

Corresponding author: Jin Chen, ebm_chenjin@126.com

610041 No. 17, Section 3, Renmin South Road, Chengdu

Tel: (028) 8542-2082

† these authors contributed equally to this work

* Corresponding author

**Table S2** Predictive accuracy comparison: PNN vs. PNN_m_

| Validation set | Model | MAE (mg/d) | Ideal-predicted percentage N (%) | | | | MSE (mg/d) |
| --- | --- | --- | --- | --- | --- | --- | --- |
|  |  |  | Overall | Low-dose group | Intermediate-dose group | High-dose group |  |
| Internal | PNN | 0.3250 | 1507 (79.1) | 0 (0.0) | 1462 (94.4) | 45 (24.3) | 0.3475 |
|  | PNN_m_ | 0.3349 | 1512(79.3) | 6(3.5) | 1460(94.3) | 46(24.9) | 0.4230 |
| External | PNN | 0.3452 | 1456 (76.4) | 2 (0.7) | 1437 (94.0) | 17 (19.8) | 0.3933 |
|  | PNN_m_ | 0.3462 | 1463(76.8) | 10(3.4) | 1435(93.9) | 18(20.9) | 0.4085 |

PNN_m_, PNN model including age, gender, weight, height, ALT and AST as mandatory input variables
